# Tuberculosis care for migrant patients in Portugal: a mixed methods study with primary healthcare providers

**DOI:** 10.1186/s12913-019-4050-0

**Published:** 2019-04-18

**Authors:** Ana Maria Tavares, Ana Cristina Garcia, Ana Gama, Ana B. Abecasis, Miguel Viveiros, Sónia Dias

**Affiliations:** 1 Global Health and Tropical Medicine, GHTM, Instituto de Higiene e Medicina Tropical, IHMT, Universidade Nova de Lisboa, UNL, Rua da Junqueira 100, 1349-008 Lisbon, Portugal; 2 Escola Nacional de Saúde Pública, Centro de Investigação em Saúde Pública, Universidade NOVA de Lisboa, Av. Padre Cruz, 1600-560 Lisbon, Portugal; 3 Departamento de Epidemiologia, Instituto Nacional de Saúde Dr. Ricardo Jorge (INSA), Av. Padre Cruz, 1649-016 Lisbon, Portugal

**Keywords:** Migrants, Tuberculosis, HIV-TB, Healthcare providers, Mixed methods research

## Abstract

**Background:**

Tuberculosis (TB) is still a major global health problem. The increasing number of cases observed among foreign-born populations contrasts with the decreasing trends observed in later years in some high-income countries. Healthcare providers are key interveners in the control of TB and HIV-TB infections. In this study, we aimed to explore the perspectives of healthcare providers working in primary care in Portugal about the provision of TB care for migrant patients with TB or HIV-TB co-infection.

**Methods:**

We applied a mixed-methods approach using an online survey and semi-structured interviews with primary healthcare providers. A total of 120 Portuguese healthcare providers participated in the survey, and 17 were interviewed. Survey and interview data were analysed applying descriptive statistics and thematic analysis, respectively.

**Results:**

Migrants’ lack of knowledge on TB disease and its symptoms was the main reason for advanced-stage presentation of cases. Their high mobility and social isolation affect adherence to treatment. The providers also listed several barriers to migrants’ access and use of TB care. The most frequently referred were limited socioeconomic resources, complex bureaucracy at the point of access and registration for healthcare services, especially for undocumented migrants, and obstacles for social protection. Providers also advocated more training initiatives on migrants’ health, social and cultural contexts, on HIV and TB integrated care, and on TB scientific update for general practitioners and nurses working at primary healthcare centres.

**Conclusions:**

Future efforts should provide measures to overcome social, economic and administrative obstacles to care for TB-infected migrants, and promote regular training initiatives for national healthcare providers in order to raise awareness and facilitate better care to culturally diverse populations with TB.

## Background

Tuberculosis is one of the top ten causes of death worldwide, causing disease in millions of people each year. In 2017, TB incidence rate in the European Region of the World Health Organization (WHO) was 30/100,000 population [1]. In the context of high-income countries, the number of TB cases has stabilized or even decreased among native-born populations in the last decade. However, among the foreign-born, the number of cases has decreased more slowly or even increased in some countries [2].

In Portugal, 1741 TB cases were notified in 2017, maintaining a trend of 5% decrease per year. The proportion of TB cases among foreign-born individuals has been increasing over the last years: 19% of the total TB cases in 2017 occurred in foreign-born individuals vs. 15.9% in 2014 [3, 4]. In 2016, the proportion of TB cases co-infected with HIV in Portugal was one of the highest within the European Union/European Economic Area [5], and 10.9% of all TB patients tested for HIV were positive [3]. Previous studies in Portugal reported a higher risk for TB infection within areas of greater density of migrants, of high prevalence of HIV/AIDS, and of poor living conditions [6]. Furthermore, the foreign-born population living in Portugal increased 6% in 2017 compared to the previous year [7], which raises particular concern in terms of TB control due to migrants’ vulnerability to TB and HIV-TB infections [8–10].

Healthcare providers play a critical role in the control of TB and HIV-TB infections, providing close support and supervision to the patients [11]. Previous research on perceptions from healthcare providers about the difficulties in the provision of care to migrant populations described structural disorganization, high workload [12–14], and lack of knowledge about migrants’ health issues [13, 15]. In addition, the lack of services adapted to the needs of migrant patients [16], their limited access to care [17, 18], the communication and cultural barriers [12, 14, 18], their low socioeconomic status, and the lack of knowledge about the functioning of healthcare services [18], have been highlighted in the literature. Previous studies addressing the Portuguese healthcare system have also explored perceptions of healthcare providers regarding the delivery of care to migrant patients [19, 20]. However, studies on TB care for migrant patients in Portugal are still lacking. In this study, we aim to explore and describe the perspectives of primary healthcare providers in Portugal and to gain an understanding about the current provision of TB care for migrant patients with TB or HIV-TB co-infection. The findings obtained in this study are expected to contribute to improve the provision of TB care for migrant patients.

## Methods

### Design and setting

A mixed-methods study was used to obtain an enriched knowledge on the healthcare providers’ perspectives and experiences about TB care for migrant patients. The quantitative approach allowed to obtain measurable evidence on providers’ perspectives, and the qualitative approach provided a deeper understanding of their perceptions [21]. The study comprised an online survey and semi-structured interviews conducted with healthcare providers working on TB care at primary care services in Portugal, namely at Chest Disease Centres (CDCs).

The National Tuberculosis Program (NTP) regulates and coordinates activities for health promotion and prevention of TB disease, and plans technical requirements for an adequate provision of care. The NTP activities are mainly facilitated in primary care, with CDCs being the main facilities involved in the routine practice [22, 23]. CDCs are healthcare units exclusively dedicated to the diagnosis and treatment of respiratory diseases and are included within Primary Healthcare Centres Clusters (PHCCs). In Portugal, TB treatment procedures are based on the current WHO recommendations [24, 25]: patients follow a daily Directly Observed Therapy (DOT) approach – daily uptake of medication under supervision of a healthcare provider – for a minimum period of 6 months [26]. The healthcare services must ensure the patient receives TB treatment at the healthcare unit closer to his residency, at home, at CDCs, other healthcare facilities, or other location agreed with the patient [27].

### Online survey

#### Sample and data collection

The online survey enrolled healthcare providers working on TB care at primary care services in Portugal, namely at CDCs, in Lisbon, North, Centre, and Alentejo Regions of Portugal. About 84% of the total foreign-born population residing in Portugal in 2017 lived in these regions (over 350 thousand foreign-born individuals) [28]. The studied regions comprised approximately 60 CDCs. In each CDC work 3–4 healthcare providers. A total of 120 healthcare providers were included in the sample, comprising nurses, medical doctors, and diagnosis and therapy technicians ^a.^^1^

The questionnaire was provided through an online survey platform. Intending to reach as much healthcare providers as possible, we sent an email to healthcare services inviting the providers to participate in the study. The providers were asked to access the web link of the survey and to complete the questionnaire. Participants were also asked to forward the email to peers. Out of a total of 185 providers who accessed the survey link, 120 completed the questionnaire. No significant differences were observed between respondents and refusals regarding sociodemographic characteristics (gender, *P* = 0.490; age, *P* = 0.693; occupation, *P* = 0.304; country of origin, *P* = 0.610; experience in TB care, *P* = 0.120; experience with migrant patients, *P* = 1.000).

The instrument included 19 multiple choice items, namely questions on sociodemographic characteristics (gender, age, country of birth), occupational history (occupation, years of professional experience in providing TB care, years of professional experience with migrant patients), practice with migrant patients (proportion of migrant patients consulted daily, stage of TB disease upon arrival to healthcare services, frequency of treatment interruption, difficulties in treatment adherence compared to nationals), barriers to access and use of TB care (cultural and language differences, migrants’ knowledge on their rights and duties, socioeconomic status, health services functioning and networking), and perceived competences and needs for training on the provision of TB care for migrant patients, and on planning strategies for treatment adherence. Reminders were sent to the healthcare services to promote participation. Data were collected from October 2016 to February 2018.

#### Data analysis

Descriptive statistical analyses were performed to describe healthcare providers’ sociodemographic characteristics, occupational history, migrants’ TB disease stage upon arrival to healthcare services, their adherence to TB treatment, perceived barriers to access and use of TB care, as well as perceived competences and training needs. Analyses were performed using SPSS Statistics v.24 software (IBM Corporation, New York, USA).

### Semi-structured interviews

#### Sample and recruitment procedure

Semi-structured interviews were conducted with healthcare providers working on TB care at primary care services, namely CDCs, in Lisbon Region. This region was inhabited by more than 182 thousand foreign-born individuals in 2017 (about 43% of the total foreign-born population in Portugal) [28].

A purposive sample of healthcare providers was obtained through snowball sampling [29]. The first contacts were provided by one researcher (ACG) from her network. The healthcare providers were then contacted via phone call or email, informed about the objectives and details of the study and invited to participate on a face-to-face interview. Interviewees were asked to identify other eligible peers. Participants included 17 healthcare providers: 11 nurses and 6 medical doctors (14 women and 3 men).

#### Data collection

Interviews were conducted from October to December 2017 in primary healthcare facilities or other public locations according to providers’ preference and availability. The interview guide included questions about the perceptions on migrants’ stage of TB disease upon arrival to healthcare services, adherence to TB treatment and related factors, barriers to access and use of TB care, and perceived competences and training needs to provide TB care to migrant patients. Interviews were audio-recorded upon participants’ agreement and informed consent. Each interview was conducted by one researcher (AMT) and lasted on average 45 min. Data collection was conducted until data saturation was reached. All participants were invited to complete a brief questionnaire for sociodemographic characterization.

#### Data analysis

The interviews were analysed using a thematic analysis approach [30]. Each interview was transcribed and analysed by one researcher (AMT) and whenever doubts emerged during analysis, a discussion was held between two researchers until consensus was reached. The initial categories for the qualitative data analysis were defined based on a preliminary literature review and descriptive analysis of the survey data. These categories further evolved and changed during the analysis of the interviews. Data were converted into segments of relevant information and concepts, then organized into the categories, and the results were analysed and interpreted. Quotes were chosen to illustrate the topics, meanings and contexts provided by the interviewees. To maintain participants’ confidentiality, the names of the interviewees and of other providers/institutions were removed from the transcripts. The interviewees were identified in the text by their occupation and their years of professional experience in TB care.

### Ethical considerations

Participation was voluntary and informed consent was obtained from all enrolled participants. All information was handled with confidentiality. Each interview was given an anonymised coding number. This study was approved by the Ethics Committees of the Regional Health Administrations of each region where the study was implemented.

## Results

The characteristics of the surveyed participants are presented in Table 1. Most participants were women (78.3%), born in Portugal (88.3%) and had 10 years or less (65.0%) of experience in TB care. There were equal proportions (46.7%) of medical doctors and nurses.

**Table 1:** Sociodemographic and professional experience characteristics of the surveyed participants

Total		
Characteristic	n	%
Gender (*N* = 120)		
Male	26	21.7
Female	94	78.3
Age (years; *N* = 120)		
20–30	8	6.6
31–40	44	36.7
41–50	30	25.0
> 50	38	31.7
Country of birth (*N* = 120)		
Portugal	106	88.3
Other	14	11.7
Occupation (*N* = 120)		
Medical Doctor	56	46.7
Nurse	56	46.7
Diagnosis and therapy technicians	8	6.6
Professional experience in TB care (years; *N* = 117)^a^		
0–10	76	65.0
11–20	17	14.5
> 20	24	20.5
Professional experience with migrant patients (years; *N* = 113)^a^		
0–10	51	45.1
11–20	30	26.5
> 20	32	28.4
Approximate amount of migrant patients consulted daily^‡^ (*N* = 120)		
None	19	15.8
Few (approx. 1 third)	95	79.2
Some (approx. half)	6	5.0
Many (more than half)	0	0.0

^a^Missing values corresponding to non-responses; ^‡^Compared with the proportion of patients consulted daily from the general population

TB tuberculosis

Of the 17 interviewees, 14 (82.4%) were women. Ages ranged from 38 to 67 years (mean 53.4 ± 2.3 years). Six providers had 10 years or less of professional experience in TB care, 4 providers had 11 to 20 years of experience and 6 providers had more than 20 years of experience. Three providers had 10 years or less of experience working with migrant patients, 7 providers had 11 to 20 years of experience and 7 providers had more than 20 years of experience.

### Migrants’ TB disease stage upon arrival to the healthcare services

More than one third of the surveyed participants considered that migrants arrive at healthcare services at an intermediate (39.6%) or advanced stage of TB disease (36.9%), while 23.5% considered that migrants arrive at an early stage. Migrants’ unawareness of TB disease and its symptoms was the main factor referred for presentation of advanced disease stage: the interviewees stated that migrants frequently neglect initial symptoms, associating, for example, cough and fatigue with smoking habits and their life style. The patients were also referred to arrive at an advanced stage of disease as a result of being tested first for other pathologies with similar initial symptoms:

*“Tuberculosis symptoms are similar to other pathologies, thus they first test to see if it is cancer, or something else. They only think of tuberculosis when they have exhausted all hypotheses ( … )”* (Nurse, 37).

Some interviewees considered that migrant-specific factors were related to presenting advanced TB disease. Among migrants who develop TB disease prior to migration, the poor living conditions and precarious healthcare services in the country of origin were indicated as responsible for the advanced disease upon arrival to services. Conversely, among settled migrants, the experience of social and economic adversities in the host country, namely poor and/or overcrowded housing, lack of knowledge on hygiene, nutritional deprivation, and limited access to care, may also contribute to an advanced stage of TB disease upon arrival to healthcare services:

*“Jobs for nationals are so few, even less for these people [migrants], which makes it difficult for them to settle, or when they do, they settle within communities of 50 to 60 people living in apartments intended for 4 ( … ) so it is a risk. Wet places, poorly ventilated, attics ( … )”* (Nurse, 38).

### Migrants’ adherence to TB treatment

The perceptions of the surveyed providers on migrants’ adherence to TB treatment are described in Table 2. Migrant-specific factors, namely the mobility of the patients and their social isolation, were considered by the interviewees as the main reasons for non-adherence to treatment. The return to the country of origin and internal mobility, with frequent changes in addresses and phone contacts, were referred to cause difficulties in follow-up, possibly leading to treatment default:

*“Since it is a long-term treatment, sometimes they are not able to stay as long as necessary and they leave treatment early. ( … ) and for us it is a little difficult, since we cannot manage this kind of treatment from the distance ( … ). They are a little constrained in their ability to stay for the time needed for the treatment.”* (Doctor, 15).

The consequent social isolation and lack of family support were referred to hamper treatment adherence and the correct follow-up of the treatment plan:

*“Without support [from family], or somebody saying ‘It is better if you take it [TB medication]’, I think it can happen [interruption of treatment]. ( … ) If the person lives alone ( … ) maybe will make more mistakes ( … ), ends up being sloppier.”* (Nurse, 12).

Some interviewees reported migrants’ difficulties in understanding the treatment plan, sometimes taking the multiple drug therapy throughout the day instead of early in the morning, as recommended. Treatment characteristics and its side effects were also referred to hinder adherence, namely the high pill burden and the long period of treatment. The side effects and the relief of symptoms after initiating treatment were also referred to favour self-perception of cure and uselessness of continuing treatment. Some patients were also referred to interrupt treatment due to incompatibilities of DOT appointments with their working hours.

**Table 2:** Perceptions on migrants’ adherence to TB treatment and on their experienced difficulties compared to nationals

Variables related to TB treatment adherence among migrant patients	Total	
	n	%
Frequency of non-adherence to TB treatment among TB-infected migrants (*N* = 88)^a^		
Rare	24	27.3
Occasional/Frequent	44	50.0
Do not know	20	22.7
Frequency of non-adherence to TB treatment among HIV-TB infected migrants (*N* = 88)^a^		
Rare	26	29.5
Occasional/Frequent	38	43.2
Do not know	24	27.3
Difficulty to complete TB treatment among TB-infected migrants compared to TB-infected nationals (*N* = 89)^a^		
Same/less difficulty	41	46.1
Higher difficulty	31	34.8
Do not know	17	19.1
Difficulty to complete TB treatment among HIV-TB infected migrants compared to HIV-TB infected nationals (*N* = 89)^a^		
Same/less difficulty	37	41.6
Higher difficulty	27	30.3
Do not know	25	28.1

^a^Missing values corresponding to non-responses

*HIV* Human Immunodeficiency virus, *TB* tuberculosis, *HIV-TB* HIV and TB co-infection

According to our quantitative findings, the participants did not consider HIV-TB infected migrants more prone to occasional/frequent non-adherence to treatment than those with only TB (43.2% vs. 50.0% among those with only TB, Table 2). In contrast, the interviewees considered that having HIV co-infection can impair treatment adherence as patients have to deal with more time-consuming medical consultations, more tests, and frequent treatment side effects. Noteworthy, some interviewees also perceived treatment adherence to be related with personal and behavioural factors, namely having a non-cooperating personality or addictions (i.e. drugs, alcohol). Moreover, some interviewees mentioned religious constraints to treatment adherence. For instance, motivating Muslim patients to comply with treatment during the fasting hours of Ramadan was particularly difficult.

Some interviewees expressed the need of a legal framework obliging patients to comply with treatment, enabling, for instance, compulsory in-patient care or deportation in cases of continuous non-adherence to treatment.

### Barriers to access and use of TB care by migrant patients

**Fig. 1:**
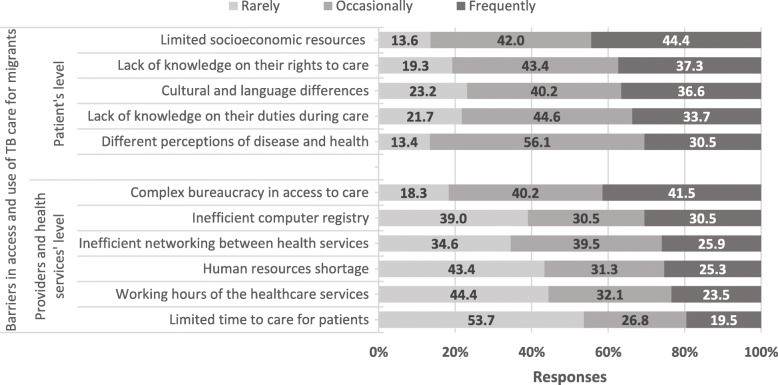
Perceived barriers for migrant patients to access and use of TB care

Barriers to migrants’ access and use of TB care perceived by the surveyed providers are represented in Fig. 1. The most frequently perceived barrier by the surveyed participants was the limited socioeconomic resources of migrant patients (44.4%; Fig. 1). In fact, half of the interviewees considered out-of-pocket payments related to transportation as a financial burden for migrant patients. This was particularly burdensome for those with HIV-TB co-infection, who also travel to the hospital for HIV medical appointments. Examples:

*“These people often have no means to buy anything. We have experienced situations in which they come to take medication and have no money for breakfast. They have no income at all. ( … ) In many cases, we had to go buy some food and make a basket of goods until the patient received the minimum subsistence”* (Nurse, 8).

*“Although everything is free of charge, merely travelling to the hospital [for HIV medical appointments] ( … ) is quite expensive, and sometimes patients have no money for food and even less for treating themselves.”* (Nurse, 9).

Also, some interviewees referred patients’ concerns related to costs with work absences and delays or with becoming unemployed while on sick leave, particularly among those with unstable non-licensed jobs. The absence of social support mechanisms preventing unemployment and loss of income lead these patients to prioritize their subsistence over their health by returning to work early:

*“When a patient with an unstable job arrives [at the CDC], we do not let him return to work because he is contagious and might infect other people. We put him on DOT but the State fails to keep his job. It’s in the law, but in practical terms, the patient loses his job, and it’s not only one case or two ( … ), they are many! ( … ) The law exists, but its application doesn’t.”* (Nurse, 30)

*“Many of these patients have a job but have never payed contributions [to social security]. So, they have to work to make a living. If they are on sick leave they do not earn any money, and cannot pay their bills ( … )”.* (Nurse, 9)

A great proportion of the surveyed providers considered the complexity of the bureaucratic procedures a frequent barrier for migrants’ access and use of TB care (41.5%; Fig. 1). Most interviewees described difficulties with the bureaucratic procedures during registration at PHCCs, the first step to access primary health care in Portugal. Whenever a patient arrived at a CDC without previous registration at a PHCC, CDC’s providers had difficulties in prescribing exams, tests and home health care. The procedures of registration, access and entitlement to co-payments were considered timely and requiring several forms from different public services. Such administrative processes were referred to be difficult for migrants, particularly for those undocumented, since many lack the required elements for the computer-based registration. Accordingly, 30.5% of the surveyed participants considered the computer-based registry a frequent barrier (Fig. 1). These constraints were even observed for undocumented migrants living in Portugal for many decades. Examples:

*“Everything is increasingly electronic and it is getting more and more complicated to register a patient. We need a VAT number, ID number ( … ).”* (Doctor, 30).

*“There is this neighbourhood where we used to provide home health care, people live here [in Portugal] for more than 30 years and are still undocumented. The doctor needed to prescribe an X-ray and she just couldn’t.”* (Nurse, 3).

One of the most frequently perceived barriers was migrants’ lack of knowledge on their rights to health care (37.3%, Fig. 1). Interviewees considered such health illiteracy as an obstacle for recently arrived migrants to navigate through the healthcare system, and was also referred as an obstacle for undocumented migrants to seek health care. Healthcare providers also stated that some migrants needlessly feared being reported to Immigration Services or being deported to their country of origin.

Cultural and language differences were less frequently perceived as barriers by the surveyed participants (Fig. 1). However, most interviewees reported existing communication barriers with migrant patients, sometimes even with migrants from other Portuguese-speaking countries due to linguistic variations of the Portuguese language. Some interviewees described barriers related to social and cultural perceptions of the disease, and stigma towards TB disease among patients and the community.

At healthcare providers’ level, workload and understaffing were referred to hamper the accomplishment of relevant tasks, including the provision of home visits to patients. The providers working on TB care at other primary care units than CDCs were referred to have an increased workload, compared to those at CDCs, due to conciliating TB care activities with other routine work tasks:

*“We feel that healthcare centres lack human resources, there are many tasks to accomplish and they have to care for a variety of situations and pathologies ( … ) sometimes they are less alert to these kind of situations [TB disease]. I know, for instance, that healthcare centres’ nurses in charge of TB care have other thousand things to do. ( … ) Here [CDC] I can provide response because this [TB care] is all I do.”* (Nurse, years of experience not provided by this participant)

At the healthcare services’ level, about half of the interviewees reported increasing barriers for the provision of social protection to TB patients and the need for more social workers placed at the healthcare units. Policy measures implemented during the Portuguese financial crisis were also referred to have reduced the support provided by the social services:

*“Our patients used to be much more supported [by the social services]. There used to be specific subsidies for TB patients ( … ). Almost all patients were entitled to a public transport pass. ( … ) Even at food level ( … ) there were institutions helping us to provide food. ( … ) Now, we are more depending on the goodwill. Do you understand?”* (Nurse, 37).

Moreover, inefficient organization of healthcare services and low problem solving capacity, were also reported. Interviewees also stated that communication problems between hospitals and primary care centres, during enrolment and referral, frequently lead to loss of follow-up.

In general, human resources constraints were not perceived as a frequent barrier by many surveyed providers (Fig. 1). However, a significantly higher proportion of providers who do not work with migrants on their daily practice (*n* = 19, Table 1), perceived human resources shortage as a frequent barrier (54.5%, *P* = 0.008, data not shown).

Some interviewees expressed the need for more psychological support services for migrant patients, especially for those with HIV-TB co-infection. More pulmonologists at primary care units, as well as adapted in-patient facilities to allow isolation of TB patients, were also suggested.

### Competences and training

Perceived competences and training needs of the surveyed providers related with providing TB care to migrant patients are described in Table 3. More than one fifth of the surveyed participants considered their competences and training insufficient to provide adequate and up to date TB care to migrants or to define a treatment adherence strategy for these patients (Table 3). Moreover, when asked about the level of agreement with the following statement: “There is a higher probability of making a mistake when providing care to migrant patients than to the general population”; one third (33.3%) of the surveyed participants agreed, 28.6% provided a neutral response and 38% disagreed (data not shown in table).

**Table 3:** Competences and training needs perceived by the healthcare providers

Total		
Perceived competences and training needs	n	%
Competences/training to provide TB care for migrant patients (*N* = 84)^a^		
Insufficient	20	23.8
Neither sufficient or insufficient	14	16.7
Sufficient	50	59.5
Competences/training to define a strategy for treatment adherence in migrant patients (*N* = 82)^a^		
Insufficient	17	20.7
Neither sufficient or insufficient	17	20.7
Sufficient	48	58.6
Relevance of receiving training about migrants’ health (*N* = 82)^a^		
Relevant	65	79.3
Moderately relevant	7	8.5
Irrelevant	10	12.2
Relevance of receiving training on strategies to improve TB treatment adherence in migrants (*N* = 82)^a^		
Relevant	64	78.0
Moderately relevant	5	6.1
Irrelevant	13	15.9

^a^Missing values corresponding to non-responses

TB tuberculosis

About half of the interviewees emphasised the relevance of increasing training initiatives for the healthcare workers dedicated to TB care, namely: actions that improve cross-cultural competencies, as well as knowledge on migrants’ social and health contexts; training on HIV and TB integrated care for healthcare providers from both specialties; and language training in order to assure providers’ proficiency in at least one foreign language. Healthcare providers working on TB care at other primary care units than CDCs were referred to have less experience with TB patients, consequently having a higher perception of TB disease as a hazard to other patients or healthcare providers. Therefore, interviewees considered important to increase updating training and raise awareness to TB disease among all providers in general.

Some interviewees also showed willingness to know more about institutions and public services to which they can refer migrant patients for support. However, scientific update and training initiatives were also considered costly, non-sponsored, and often restricted to certain professional groups — usually the superiors rather than routine service providers in close contact with the patients.

Noteworthy, a significantly higher proportion of providers who do not work with migrants on a daily basis (*n* = 19, Table 1) considered training on migrants’ health (44.4%, *P* = 0.023), and on strategies to improve treatment adherence in migrants (44.4%, *P* = 0.028) as irrelevant (data not shown in table).

## Discussion

This mixed-methods study allowed to gain insight into the perspectives of primary healthcare providers on current provision of TB care for migrants in Portugal. Our findings highlighted several factors hampering the provision of TB care to these populations that need to be addressed. These included factors related to the specific context of migration and also factors affecting the provision of TB care to all patients in general.

Over a third of the surveyed providers referred that migrants entering the healthcare system are frequently in an advanced stage of TB disease, mainly due to their unawareness of the disease and its symptoms. This is in line with the existing literature showing that limited knowledge and understanding about TB causes, symptoms, modes of transmission and treatment deeply influences migrants’ health-seeking behaviours [31]. Migrant patients may have differing values, beliefs and concepts of disease and health based on their ethnic and cultural background [32]. The preventive care culture varies from country to country, being sometimes weak or even absent in the countries of origin of many migrants [32]. These cultural factors can shape how people understand signs and symptoms and perceive healthcare needs, delaying care-seeking behaviour [20]. Such delays in prompt TB diagnosis and/or treatment can contribute to onwards transmission of TB within migrant communities [33]. Health campaigns targeted to migrant communities should be promoted to raise awareness towards TB disease and increase health literacy.

In our study, the healthcare providers referred that many migrants struggle to comply with TB treatment, mainly due to their frequent mobility and the absence of family support. Modern migration patterns involve recurrent travels between origin and destination countries, which can increase risk of treatment interruption [31]. This is particularly worrying, given that suboptimal adherence to TB treatment may cause drug resistance [33] and consequently compromise treatment and disease outcomes. In addition, previous studies described the importance of social, emotional and financial support from families and communities on treatment adherence and good treatment outcomes [34–37]. Our findings reinforce that optimal adherence to treatment among migrant patients requires increasing support from family and community members in the treatment process.

Limited socioeconomic resources of migrant patients were referred by the healthcare providers as the main barrier to TB care. Previous studies in Portugal have reported lower access and use of healthcare services among migrants in disadvantaged socioeconomic situation [38, 39]. Other studies on TB care concluded that even when diagnosis and treatment are free of charge, indirect costs, namely related to transportation, loss of income, and productivity, hinder prompt uptake of treatment [37]. Our findings also indicate that the effect of economic constraints for migrants may be exacerbated by the lack of social protection while on treatment. Moreover, political measures taken in the context of the financial crisis in Portugal were referred to impair mechanisms of social protection that used to be available for TB patients with low socioeconomic status. Budget-balancing measures introduced into the social protection system during the financial crisis have tightened the eligibility for social assistance, unemployment benefit, and other protection mechanisms, increasing poverty rates [40]. These constraints surely affected migrant populations disproportionally as they are often socioeconomically more vulnerable [41]. Increasing social protection spending can contribute to reduce loss of income and poverty, improving access and use of TB care and consequently clinical outcomes [42, 43]. In this work, we propose that social protection for migrants must be strengthened and linked to healthcare services. Future political measures aiming to improve TB care efficacy among migrant populations must also target financial support to migrant patients with low socioeconomic status.

In our study, another significant barrier was the complexity of bureaucratic procedures required for migrants to access primary care services, particularly for those with undocumented status. Portugal has been recognized for implementing migrant-friendly policies [41]. By law, migrants in Portugal have the same access to the healthcare system as Portuguese citizens once they obtain a residence permit. Free access to health care is guaranteed in situations of urgent and vital care, communicable diseases, among others. Fees exemption is also granted in situations of public health threat, such as TB or HIV, including for undocumented migrants [41, 44]. However, our study indicates a discrepancy between legislation and its application to health practice. As observed in our study, despite of being entitled to care, some migrants are unaware of their rights and some also fear being deported or reported to Immigration Services, leading to underuse of TB care. These findings are consistent with other studies [45].

Appropriate access to TB care, regardless of the legal status, is crucial for individual’s and community’s health [33]. With this in mind, barriers should be reduced in order to improve access to the healthcare system and thus ensure prompt diagnosis and treatment. Specifically, the information system should be simplified in order to enable migrants’ registration in a more straightforward and flexible manner and, especially, to ensure compliance with the current legislation. These measures might also improve general patient satisfaction and healthcare providers´ efficiency.

In this study, many healthcare providers perceived having limited competences and training about the social, cultural and health context of migrant patients. In addition, a considerable proportion of the surveyed participants reported low self-confidence in providing TB care for migrants, which can be partially explained by the low number of migrant patients consulted daily. Providers also referred that colleagues working at primary care units other than CDCs often face high workload, conciliating TB care with other routine tasks, and that they could benefit from further training in TB care. Our findings suggest that training on provision of TB care to culturally diverse populations should be supported in the future, in order to enhance optimal performance of healthcare providers [12, 46].

Reaching the global TB targets aligned with the 2030 Agenda for Sustainable Development and as part of the End TB Strategy requires universal health coverage of essential health services, and social protection mechanisms to prevent TB patients from suffering catastrophic costs [47]. The WHO and the International Organization for Migration proposed actions that support personnel cultural competence, culturally sensitive healthcare services, including HIV-TB management, and the implementation of policies that aim to improve migrants’ access to health services and to eliminate legal and administrative barriers [31]. Although political measures in Portugal have attempted to follow those recommendations, our study revealed that several barriers to TB care still prevail for migrants. These barriers may compromise migrants´ health, as well as ongoing public health control measures [48–50], and therefore should be mitigated in the future.

We acknowledge some limitations of our study. The limited response rate may have possibly introduced a nonresponse bias. However, similar reduced response rates are commonly observed in studies involving healthcare providers, particularly medical doctors [51]. Likewise, in a recent systematic review on response rates of general practitioners from primary care in Portugal, an average response rate of 56% (95%CI 47–64%) was observed [52]. Furthermore, no significant differences were observed between respondents and refusals concerning their sociodemographic characteristics. Another limitation was the inability to include providers from all regions of Portugal. However, it was possible to include providers from regions in which overall inhabit about 84% of the total foreign-born population [28]. Additionally, we also acknowledge the possibility that providers who agreed to participate in the study shared a particular interest in this research topic, introducing a self-selection bias. Nevertheless, considering the limited number of healthcare providers dedicated to TB care in some of the enrolled primary care settings, the sampling methods used in this study allowed us to reach these providers and to obtain their valuable views.

A strength of this study was the use of a mixed methods approach, which allowed to gain a deeper, broader and richer understanding of the providers’ perceptions, compared with either quantitative or qualitative methods alone [53]. This type of approach was particularly valuable for our study considering the reduced number of providers dedicated to TB in primary care, and, most of all, allowed us to collect relevant perspectives from those who are key interveners in the control of TB disease. Moreover, the anonymity and confidentiality guaranteed throughout the study rendered the necessary comfort to participants go deeper into their opinions.

The main relevance of this study is its contribution to increase scientific evidence on the underexplored theme of TB care for migrant patients in the Portuguese context, identifying existing obstacles and highlighting targets for future improvement measures.

## Conclusions

This study was, to our knowledge, the first to explore the difficulties faced by TB-infected migrants in Portugal to seek TB care, comply with TB treatment and access and use healthcare services for TB care. Such insight was gained through the perspectives of healthcare providers in close contact with the patients. Our findings suggest that future efforts should focus on measures to overcome social, economic and administrative obstacles to care for TB-infected migrants. Training initiatives for healthcare providers should also be promoted in order to improve TB care to culturally diverse populations.

## Abbreviations

ABA: Ana B. Abecasis
ACG: Ana Cristina Garcia
AIDS: Acquired Immunodeficiency Syndrome
AMT: Ana Maria Tavares
CDC(s): Chest Disease Centre(s)
DOT: Directly Observed Therapy
HIV: Human Immunodeficiency Virus
HIV-TB: HIV and Tuberculosis co-infection
NTP: National TB Programme
PHCC(s): Primary Healthcare Centres Cluster(s)
SD: Sónia Dias
TB: Tuberculosis
WHO: World Health Organization

## Footnotes

1 Provider who uses scientifically based techniques for health promotion aimed at prevention and diagnosis. Develops activities with autonomy and complementarity with other professional groups. Examples: Clinical and Public Health Analysis Technician; Radiology Technician [54].
